# Brown adipose tissue enhances exercise performance and healthful longevity

**DOI:** 10.18632/aging.206179

**Published:** 2024-12-18

**Authors:** Dorothy E. Vatner, Jie Zhang, Stephen F. Vatner

**Affiliations:** 1 Department of Cell Biology and Molecular Medicine, Rutgers New Jersey Medical School, Newark, NJ 07103, USA

**Keywords:** brown adipose tissue, white adipose tissue, healthful longevity, exercise, regulator of G protein signaling 14

## Abstract

Brown adipose tissue (BAT), a major subtypes of adipose tissues, is known for thermogenesis and promoting healthful longevity. Our hypothesis is that BAT protects against impaired healthful longevity, i.e., obesity, diabetes, cardiovascular disorders, cancer, Alzheimer’s disease, and reduced exercise tolerance. While most prior studies have shown that exercise regulates BAT activation and improves BAT density, relatively few have shown that BAT increases exercise performance. In contrast, our recent studies with the regulator of G protein signaling 14 (RGS14) knockout (KO) model of extended longevity showed that it enhances exercise performance, mediated by its more potent BAT, compared with BAT from wild type mice. For example, when the BAT from RGS14 KO mice is transplanted to WT mice, their exercise capacity is enhanced at 3 days after BAT transplantation, whereas BAT transplantation from WT to WT mice increased exercise performance, but only at 8 weeks after transplantation. The goal of this research perspective is to review the role of BAT in mediating healthful longevity, specifically exercise capacity. In view of the ability of BAT to mediate healthful longevity and enhance exercise performance, it is likely that a pharmaceutical analog of BAT will become a novel therapeutic modality.

## White adipose tissue (WAT) vs. brown adipose tissue (BAT)

There are two major subtypes of adipose tissue, i.e., WAT and BAT, each with distinct functions, cellular structures, and origins. WAT originates from mesenchymal stem cells during development and consists of large, unilocular adipocytes. These cells contain a single lipid droplet and are found in areas like the abdomen, thighs, and around internal organs [1]. WAT functions primarily as an energy reservoir, storing excess calories in the form of triglycerides, but also plays a role in impairing healthful longevity by increasing obesity and its related metabolic disorders, such as insulin resistance, type 2 diabetes, and cardiovascular diseases [2, 3].

### Brown adipose tissue (BAT)

In marked contrast to WAT, BAT is becoming increasingly attributed to healthful aging. Brown adipocytes are smaller in size than white adipocytes with lipid droplets surrounding the nucleus. Brown adipocytes have mitochondria dispersed between the droplets, which give these cells their brown appearance. BAT is mainly located in the interscapular space of mice, and in humans it is found in the interscapular, supraclavicular, suprarenal, and para-aortic spaces. BAT originates from a lineage closely related to skeletal muscle cells, also derived from mesenchymal stem cells. BAT consists of smaller adipocytes with mitochondria, which produce heat through uncoupling protein 1 (UCP1) [4], resulting in the release of heat [1, 5–8]. This provides the basis for the major role for BAT, thermogenesis, as a mechanism to maintain body temperature by helping maintain body temperature in cold environments. This function is especially critical in newborns, who have a higher proportion of BAT, but it persists in adults in smaller amounts, especially around the neck and shoulders [9]. Changes in BAT with aging reduce its effects on thermogenesis [10]. BAT is activated by norepinephrine, a hormone released during cold exposure and sympathetic nervous system activation. Norepinephrine binds to β-adrenergic receptors on brown adipocytes, stimulating the production of UCP1, which uncouples oxidative phosphorylation from ATP synthesis, allowing energy to be dissipated as heat [5]. Additionally, thyroid hormones are important for BAT activation and thermogenesis, enhancing the tissue’s ability to burn fat [11].

In addition, UCP1-independent thermogenic pathways have been found in beige adipocytes and muscles [12]. BAT also functions as a metabolic sink by oxidizing glucose and lipids, which produces heat. Since BAT burns calories to produce heat, its activation promotes weight loss, and enhances insulin sensitivity [13], which have therapeutic potential for obesity and diabetes [14]. BAT has also been shown to mediate healthful longevity [8, 15].

## BAT mediating healthful longevity

Studies have indicated that aging reduces BAT activity leading to thermal dysregulation and energy imbalance [16–18]. However, the effects of age on BAT mass have been inconsistent [19–21]. Some studies have reported that aging increases the amount of BAT [19, 20], whereas one study reported no change in BAT mass in rodents [21]. In addition, beige adipocyte formation declines with aging which may be caused by changes in the adipose tissue microenvironment [16, 22].

More recently, interest has extended to BAT’s role in mediating healthful aging, primarily from data in genetically altered mouse models. It has been reported that the enhanced BAT activity/function mediate healthful longevity in several longevity mouse models, e.g., protection against obesity [23, 24], diabetes [23, 24], cardiovascular disorders [25–27], cancer [28–32], Alzheimer’s Disease [33], stroke [34, 35], exercise intolerance [36], and reduced blood flow [36–38].

In addition, there are models with enhanced BAT function or extra BAT amount by BAT transplantation that exhibit aspects of healthful longevity. WT mice receiving BAT from another WT BAT mouse exhibit improved exercise capacity, protection against obesity and diabetes and cancer [36, 39–41]. It has also been suggested that low levels of BAT in humans are associated with obesity and glucose intolerance, whereas those with higher BAT levels maintain lower body weight and more healthful aging [42].

**Figure 1:**
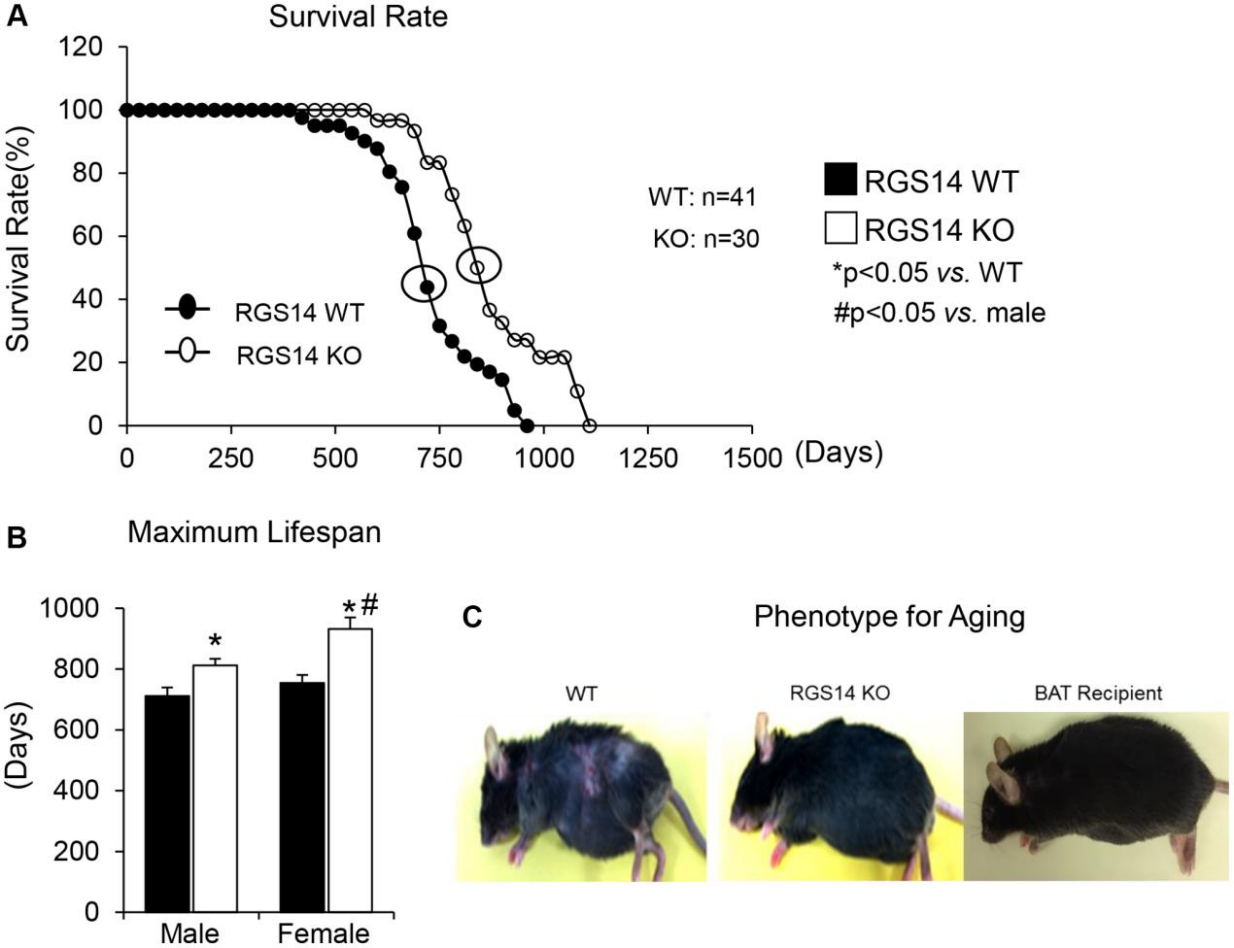
**RGS14 KO model of longevity.** (**A**) Kaplan-Meier survival curves for RGS14 KO and WT mice showed significantly augmented survival in RGS14 KO mice. (**B**) Maximum lifespan was significantly greater in RGS14 KO mice than in WT mice for both males and females. In addition, medium and maximum lifespan were greater in female RGS14 KO mice than in male RGS14 KO mice. (**C**) Furthermore, 24 month old RGS14 KO mice did not show the aging phenotype normally present in WT mice of similar age, including body atrophy, loss of hair and greying of fur color. In support of the key role of BAT in aging, old WT RGS14 KO BAT recipient mice, which had BAT transplanted at 3–4 months of age, had the appearance of healthful aging similar to the old RGS14 KO mice. A representative example of each is shown in panel C. For median lifespan analysis, a Mood’s Median Test, was used to determine differences in median lifespan. A Student’s *t*-test was used to test differences in maximum lifespan. ^*^p < 0.05 vs. WT, ^#^*p* < 0.05 vs. male. Reprinted from Vatner DE, et al Aging Cell. 2018; 17(4).

**Figure 2:**
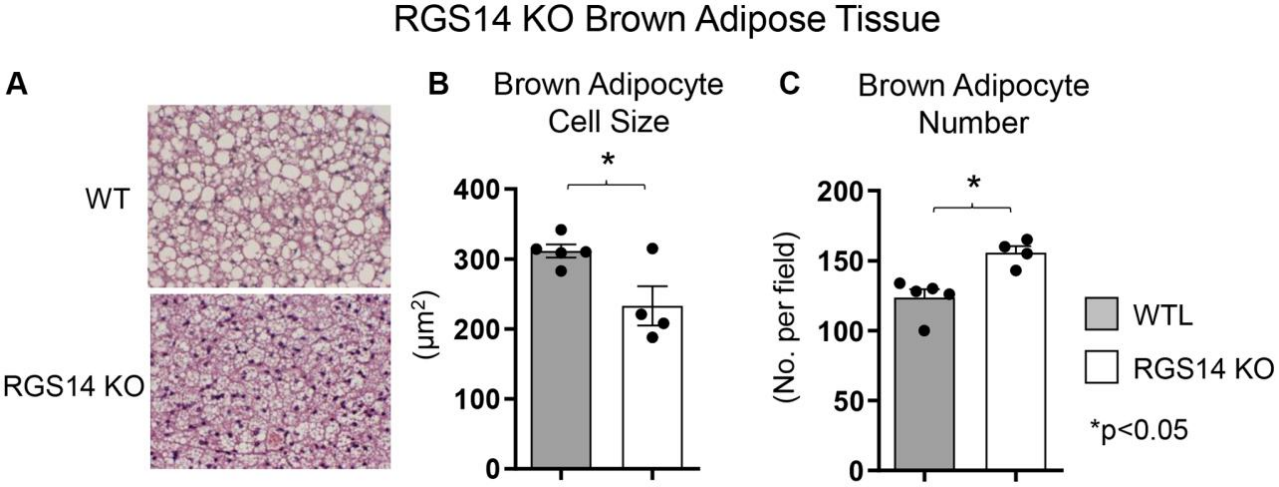
**Increased BAT cell numbers in RGS14 KO mice.** RGS14 KO mice exhibited smaller brown adipocytes (**A**, **B**), and increased number of brown adipocytes (**A**, **C**) than WT control mice. ^*^p < 0.05. Reprinted from Vatner DE, et al Aging Cell. 2023; 22(4).

One example of a genetic model demonstrating that BAT enhances exercise performance is that of RGS14 KO mice, a healthful lifespan model, mediated by increased BAT [8]. Survival is significantly enhanced in the RGS14 KO mouse, with females living longer than males, similar to human data (Figure 1A, 1B). In addition, the old RGS14 KO mice do not show the phenotype of aging WT mice, i.e., body atrophy, loss of hair and greying of hair color (Figure 1C). These features of healthy aging can be recapitulated in WT mice with transplants of BAT from RGS14 KO mice at a young age (Figure 1C). The RGS14 KO mice have increased density of BAT with smaller BAT cell size (Figure 2).

## BAT and exercise

### Exercise regulating BAT

Enhanced exercise capacity is not only a feature of healthful aging, but also is a therapy for aging patients and patients with cardiovascular disease. Exercise is a healthy way to reduce body weight by activating the sympathetic nervous system, accelerating the decomposition of fat, and promoting the utilization and consumption of energy in skeletal muscle [43–45]. During aging, it is known that progressive loss of exercise capacity relates to loss of skeletal muscle mass and tissue function [46]. Decreased muscle mitochondrial function contributes to the loss of skeletal muscle function during aging [47–51]. Regular exercise or exercise training protects against decreased muscle function during aging [52, 53], frailty status [54, 55], and neurodegeneration [56, 57].

Numerous studies have suggested that exercise may play a role in regulating BAT activation. Exercise boosts the expression of UCP1 and genes associated with mitochondria biogenesis, thereby improving BAT’s heat production capacity [5]. For instance, swim training in rodents over six to eight weeks increased UCP1 protein levels in BAT [58, 59]. Similarly, treadmill exercise in rodents for 6–8 weeks increased BAT activity and cytochrome oxidase activity, oxygen consumption rates and BAT-specific gene markers, e.g., UCP1, FGF21, and PGC1α [60, 61]. However, conflicting findings also exist, with some studies suggesting that exercise may reduce the thermogenic effect of BAT. In rats, six to eight weeks of moderate-intensity treadmill exercise led to decreased UCP1 expression in BAT and a reduction in total BAT mass [62, 63]. Human studies also showed inconsistent results regarding the role of exercise on BAT modulation, with some indicating that high-intensity physical activities can increase BAT density [64], while others reporting that exercise decreases glucose uptake in BAT [65–67]. While most of these studies have shown that exercise increases BAT, relatively few have shown that BAT increases exercise performance [36, 41, 68].

### BAT regulating exercise

**Figure 3:**
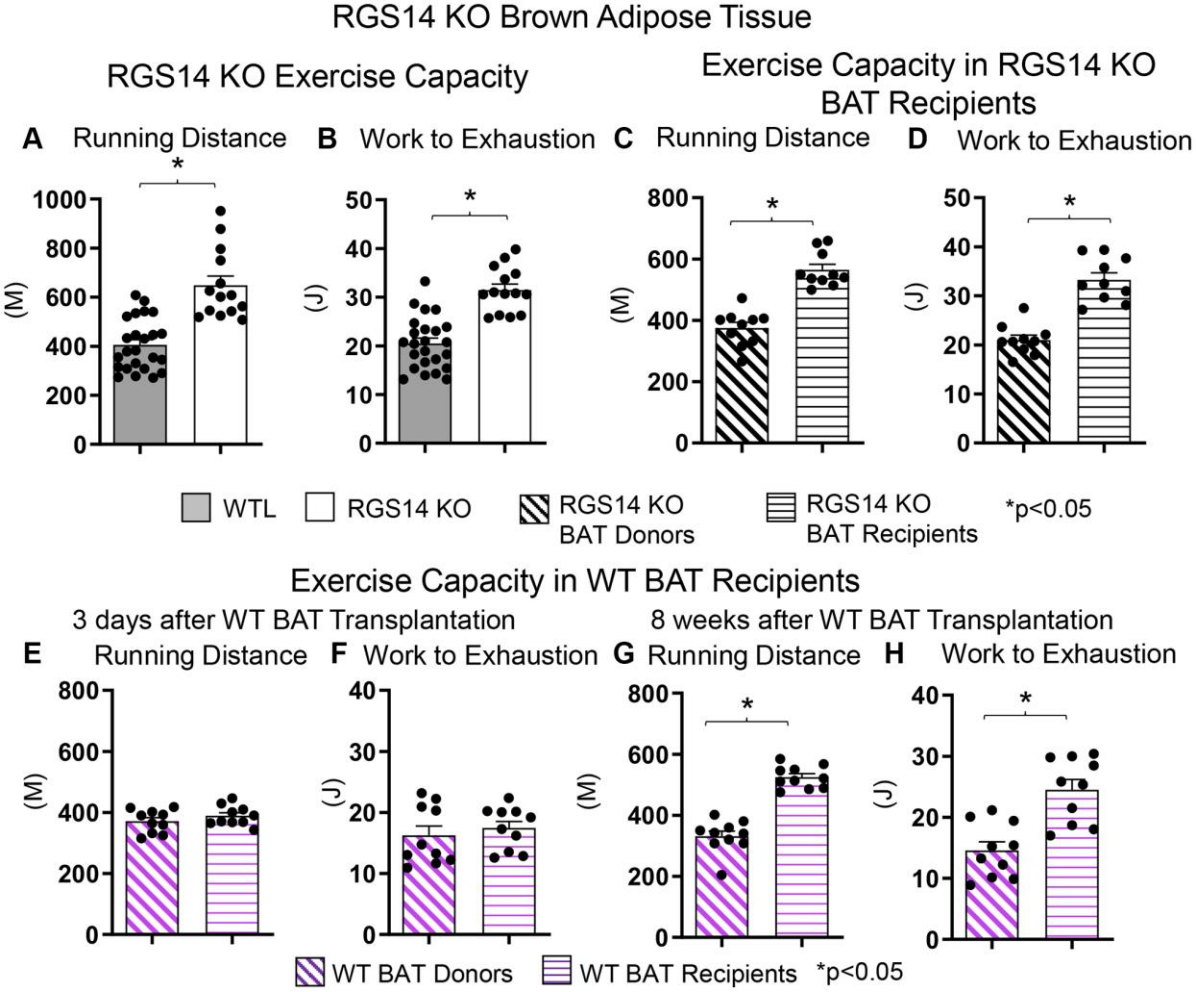
**Increased exercise capacity in RGS14 KO mice.** RGS14 KO mice ran longer distances (**A**) with increased work to exhaustion (**B**) compared to WT littermates. BAT transplantation from RGS14 KO mice to WT mice led to a reversal of phenotype, such that RGS14 KO BAT recipients exhibited improved running distance (**C**) and greater work to exhaustion (**D**) compared to RGS14 KO BAT donors, at 3 days after RGS14 KO BAT transplantation. In contrast, there was no improvement in running distance and work to exhaustion at 3 days after transplantation of BAT from C57BL6/J WT mice to other C57BL6/J WT mice (**E**, **F**). It required 8 weeks to achieve enhanced running distance and work to exhaustion in C57BI/6J WT mice with BAT transplantation from other C57BL6/J WT mice (**G**, **H**). ^*^p < 0.05. Reprinted from Vatner DE, et al Aging Cell. 2023; 22(4).

There are fewer studies examining the role of BAT regulating exercise than exercise regulating BAT. One example of a genetic model demonstrating that BAT can enhance exercise performance is that of RGS14 KO mice, a healthful lifespan model, mediated by increased BAT 8. Whereas the RGS14 KO mouse exhibits increased exercise capacity, similarly, RGS14 KO BAT transplanted to WT mice demonstrate an increased maximal running distance and work to exhaustion, which corresponds to the enhanced exercise capacity of RGS14 KO mice (Figure 3A–3D). The enhanced exercise capacity observed in WT mice with RGS14 KO BAT transplants was observed at three days after BAT transplantation, whereas BAT transplantation from WT to WT mice also resulted in increased exercise performance, not at 3 days, but only at 8 weeks after transplantation [36] (Figure 3E–3H). Another study found that BAT transplantation prevents the impaired glucose tolerance, the increase in left ventricle mass, and exercise intolerance following myocardial infarction [41]. In addition, BAT transplantation significantly reversed the reduction in physical activity in high fat diet-fed mice [68].

**Figure 4:**
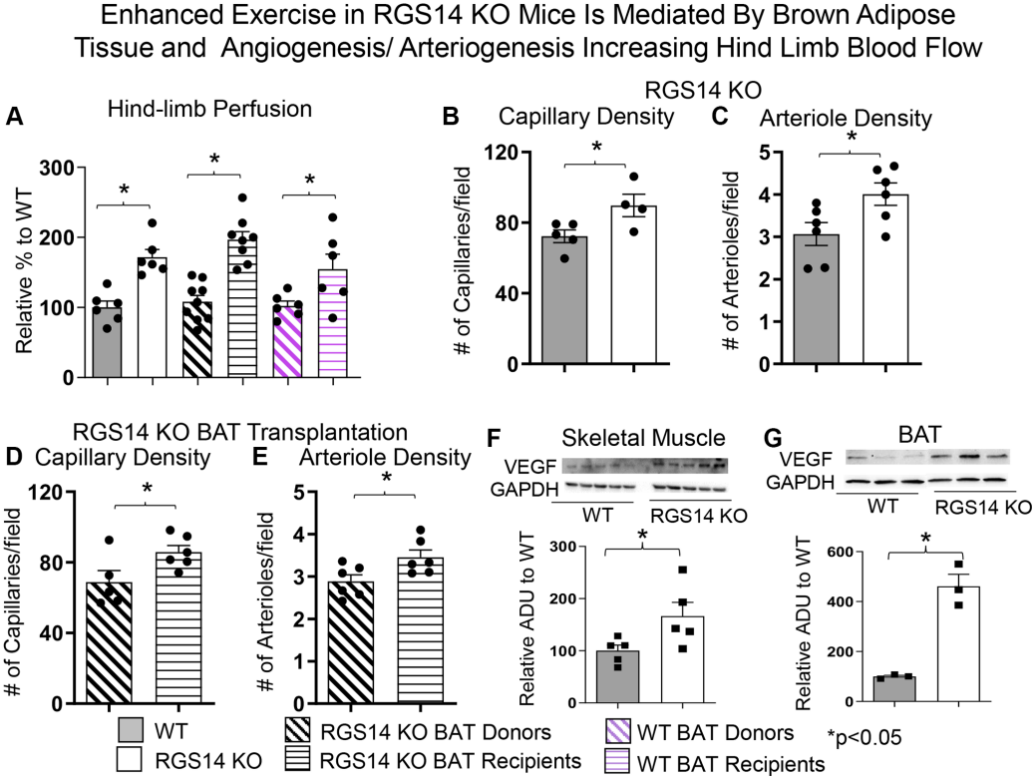
**Enhanced exercise by RGS14 KO mice is mediated by BAT and angiogenesis/arteriogenesis increasing hindlimb blood flow.** Non-linear contrast imaging was used to measure hindlimb blood flow. The average data are presented as % of WT perfusion, which is represented as 100% (**A**). Hindlimb blood flow was higher in RGS14 KO mice compared to WT mice, and higher in WT mice that received RGS14 KO BAT, at 3 days after transplantation (**A**, **B**), while RGS14 KO BAT donors lost their enhanced hindlimb perfusion, with results similar to WT mice (**A**). WT BAT recipients showed greater hindlimb perfusion at 8 weeks after transplantation of BAT from C57BL6/J WT mice to other C57BL6/J WT mice (**A**). Angiogenesis (reflected by capillary density) and arteriogenesis (reflected by arteriole density) were both increased in skeletal muscle of RGS14 KO mice (**B**, **C**) and RGS14 KO BAT recipients (**D**, **E**), which correlated with increased VEGF in skeletal muscle (**F**) and BAT (**G**). Increased angiogenesis (**D**) and arteriogenesis (**E**) were not observed in RGS14 KO BAT donors. Results are expressed as Mean ± SEM, ^*^p < 0.05. Reprinted from Vatner DE, et al Aging Cell. 2023; 22(4).

**Figure 5:**
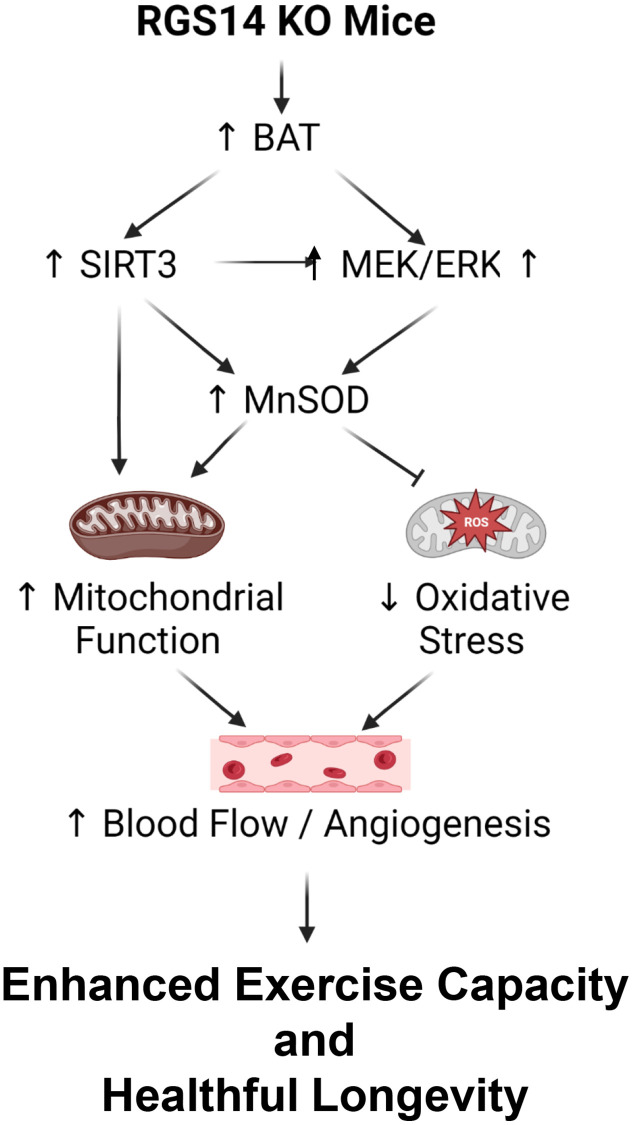
**Mechanisms mediating enhanced exercise capacity in RGS14 KO and its uniquely powerful BAT.** Multiple mechanisms mediated the enhanced exercise capacity in RGS14 KO mice. The most important mechanism is BAT, which mediates SIRT3, MnSOD, MEK/ERK and VEGF pathways. These mechanisms regulate exercise capacity by improved mitochondrial function, protection against oxidative stress and improved blood flow/angiogenesis. Reprinted from Vatner DE, et al Aging Cell. 2023; 22(4).

There are multiple mechanisms mediating the enhanced exercise capacity in RGS14 KO mice. Most importantly, RGS14 KO mice demonstrate increased hind limb perfusion (Figure 4A), also found in RGS14 KO BAT recipients, but lost in RGS14 KO BAT donors (Figure 4A) and increased capillary and arteriolar density (Figure 4B, 4C). The increase capillary and arteriolar density was also observed in RGS14 KO BAT recipients and lost in RGS14 KO BAT donors (Figure 4D, 4E). A key mechanism mediating the increased perfusion and vascular density was VEGF (Figure 4F), observed in RGS14 KO skeletal muscle and BAT (Figure 4G). At the cellular level, the mechanisms mediating the RGS14 KO and RGS14 KO BAT enhanced exercise capacity include increases in SIRT3, MEK/ERK, MnSOD, and mitochondrial function, which decrease oxidative stress. All these mechanisms are illustrated in Figure 5.

In view of the data demonstrating that the RGS14 KO mouse exhibits many features of healthful aging, one of which is enhanced exercise capacity, mediated by BAT, and that RGS14 KO BAT is more powerful than WT BAT, it becomes increasingly important to develop a pharmacological analog of RGS14 KO BAT that can be translated to the clinics to promote enhanced exercise capacity and healthful aging in patients.
